# Using Digital Communication Technology to Improve Neonatal Care: Two-Part Explorative Needs Assessment

**DOI:** 10.2196/38435

**Published:** 2023-02-07

**Authors:** Kim Tenfelde, Marjolijn Antheunis, Emiel Krahmer, Jan Erik Bunt

**Affiliations:** 1 Department of Communication and Cognition Tilburg University Tilburg Netherlands; 2 Department of Pediatrics Elisabeth-Tweesteden Ziekenhuis Tilburg Netherlands

**Keywords:** mobile health, mHealth, physician-patient communication, questions asking, needs assessment, explorative, mobile phone

## Abstract

**Background:**

The birth of a premature infant and subsequent hospitalization are stressful events for parents. Therefore, accurate and easy-to-understand communication between parents and health care professionals is crucial during this period. Mobile health (mHealth) technologies have the potential to improve communication with parents at any time and place and possibly reduce their stress.

**Objective:**

We aimed to conduct a 2-part explorative needs assessment in which the interaction between the pediatrician and parents was examined along with their digital communication technology needs and interest in an mHealth app with the aim of improving interpersonal communication and information exchange.

**Methods:**

Overall, 19 consultations between parents of preterm infants and pediatricians were observed to determine which themes are discussed the most and the number of questions asked. Afterward, the parents and the pediatrician were interviewed to evaluate the process of communication and gauge their ideas about a neonatal communication mHealth app.

**Results:**

The observations revealed the following most prevalent themes: breastfeeding, criteria for discharge, medication, and parents’ personal life. Interview data showed that the parents were satisfied with the communication with their pediatrician. Furthermore, both parents and pediatricians expected that a neonatal mHealth app could further improve the communication process and the hospital stay. Parents valued app features such as asking questions, growth graphs, a diary function, hospital-specific information, and medical rounds reports.

**Conclusions:**

Both parents of hospitalized preterm infants and pediatricians expect that the hypothetical mHealth app has the potential to cater to the most prevalent themes and improve communication and information exchange. Recommendations for developing such an app and its possible features are also discussed. On the basis of these promising results, it is suggested to further develop and study the effects of the mHealth app together with all stakeholders.

## Introduction

### Background

The birth of a preterm infant has an incredible impact on the family. Parents suddenly have to adjust to unfamiliar surroundings, cope with the infant’s uncertain survival and outcome, adjust to the hospital setting, learn new medical vocabulary, and eventually care for a vulnerable infant at home [1,2]. These factors are a source of stress, uncertainty, and fear [3]. For parents, adequate communication and information is one of the most important needs for coping with these factors and successfully navigating through the emotional hospital stay [1,4-6].

Most preterm infants are hospitalized for weeks to months in neonatal wards. During this period, parents typically receive daily medical updates from nurses and pediatric residents. In addition, patients have a weekly update consultation with their principal pediatrician. The principal pediatrician has responsibility over medical decisions during hospital stay and maintains long-term contact with the parents and patient. Although the information exchange between parents and pediatricians has improved considerably over the last few years [7,8], recent research has also revealed that the information exchange is not yet in its most optimal form. Parents still yearn for more information and do not always feel comfortable asking questions [9]. To gain a sense of control, parents often seek information from external sources, such as educational materials, the internet, and mobile health (mHealth) apps [4,8-11].

It has been suggested that mHealth technologies have the potential to further improve communication and interpersonal relationships in neonatal units [12-14]. The use of mHealth apps is gaining popularity owing to their ubiquitous availability and accessible use. In addition, practically all young parents now own a smartphone and have considerable skills in using the device [14-16].

The number of mHealth apps developed to improve communication and medical conditions is increasing, demonstrating the demand for such apps in society. A literature review by Richardson et al [12] found 18 different neonatal mHealth apps in both the Apple App and Google Play store. In general, these apps provide information on premature births, advice or tips, and monitoring of infants’ data. However, only 1 of these 18 mHealth apps (MyPreemie) was supported by scientific research [17]. There is hardly any scientific knowledge regarding the development and evaluation of neonatal mHealth apps [18,19].

To successfully build mHealth apps and have a fair possibility of successfully implementing a potential mHealth solution, it is important to involve all stakeholders during the development process [20]. Involving mHealth users from the beginning of the process, from conceptualization of the ideas to development to evaluations, enhances app adoption [19,20]. Unfortunately, potential users have rarely been involved.

Furthermore, efforts to develop technological interventions for neonatal care and guiding interpersonal communication in the neonatal ward are hampered by a distinct lack of scientific data on how parent-provider interactions organically unfold [18]. Little is known about the actual course, the contents, the themes parents find important to discuss, and the effectiveness of the interaction and information exchange between parents and medical caregivers during hospitalization [18]. Therefore, it is important to also understand communication in the neonatal ward when developing an mHealth solution [18].

### Study Aims

In this study, a 2-part exploratory needs assessment was conducted. The first part of this study (research question [RQ] 1, 2, and 3) consisted of observing, examining, and evaluating the weekly update consultation held between the principal pediatrician and the parents. The themes that were discussed and questions from parents and pediatricians were explored. This information provided a solid base for the second part of this study (RQ 3, 4, and 5). In part 2, we begin by outlining and examining interest in the idea of an mHealth app. We propose an mHealth app that can be used by parents to store themes, concerns, and questions for discussion with the pediatrician for the subsequent consultation, given that patients or parents of patients frequently forget questions they would like to ask during a consultation owing to emotional overload [4,9]. In addition, professionals are often confronted with questions that they cannot immediately answer during the consultation. Using the same app, the pediatrician could read these themes, concerns, and questions beforehand and prepare for the consultation. We speculate that using such an app could increase patient and professional satisfaction and improve communication and information exchange [21]. To investigate our aims, we propose the following RQs:

- RQ1: Which themes are discussed between the parents and pediatrician during the weekly consultation and who initiates these themes?
- RQ2: How many questions do parents and pediatricians ask during consultations?
- RQ3: How do the parents evaluate the consultation?
- RQ4: What are the parents’ opinions regarding an mHealth app?
- RQ5: Which features would the parents value in a neonatal mHealth app?
- RQ6: What are the pediatricians’ opinions regarding an mHealth app?

## Methods

### Sample

In total, 20 consultations of parents with the pediatrician were observed. Each parent-couple (PC) was observed once, and 7 individual pediatricians were enrolled. One observation was excluded from the eventual data set owing to a missing survey, resulting in a final data set of 19 consultations. Research has shown that qualitative interview studies with at least 13 samples are robust [22]. In addition, we noted that data saturation was reached after 13 consultations. Therefore, this sample size was sufficient. The consultations lasted between 10 and 45 minutes. In total, 10 consultations were first-time consultations for the parents and 9 were second or third consultations. Mothers (mean age 32.1, SD 4.9 years) were always present (n*=*19) and fathers (mean age 32.7, SD 4.6 years) were present in 15 consultations.

The 19 mothers had a total of 22 preterm infants (3 twins). The postnatal age of the preterm infants ranged from 5 to 59 (mean 17.7, SD 15) days. For 11 parents, their premature newborn was their first child; for 6 parents, the newborn was their second child; and for 2 parents the newborn was their fourth child. In 3 cases, the parents had premature twins. All infants were admitted to a level II neonatal intensive care unit (NICU) during the study. Level II NICUs provide special high or medium care for moderately ill infants [23]. Twelve of the 22 infants had previously been admitted to a level III and IV NICU where they had been critically ill at that time. Ten infants had been admitted to the level II NICU only. Table 1 presents the characteristics of the sample.

**Table 1:** Demographic and obstetric characteristics of the sample.

Characteristics						Values
**Parent details (n=35)**						
	**Nationality** **, n (%)**					
		Dutch			33 (94.2)	
		Moroccan			1 (2.9)	
		Turkish			1 (2.9)	
	**Education level** **, n (%)**					
		High school			9 (25.7)	
		Intermediate vocational education			14 (40)	
		Higher vocational education			12 (34.3)	
		University			0 (0)	
**Birth details (n=22)**						
	**Gestational age (weeks), n (%)**					
		27-28			4 (18.2)	
		29-30			3 (9.1)	
		31-32			7 (31.8)	
		33-34			6 (27.3)	
		35			2 (9.1)	
	**NICU^a^ admission (age in days)**					
		**Level III or IV NICU transfers**				
			Value, n (%)	12 (54.5)		
			Value, mean (SD)	23.8 (18.3)		
		**Level II NICU**				
			Value, n (%)	10 (45.5)		
			Value, mean (SD)	11 (6)		

^a^NICU: neonatal intensive care unit.

### Materials and Procedure

This study was conducted from March to July 2019. In total, 17 consultations were conducted at the Elisabeth TweeSteden Hospital, Tilburg, the Netherlands. Two consultations were conducted at the Jeroen Bosch Hospital, Den Bosch, the Netherlands. These 2 level II NICUs were 20 km apart, worked closely together, and followed the same medical and conversational policy. Parents and pediatricians received oral and written information regarding the study. All participants who were approached were willing to participate in the study and provided written informed consent. The observations were audio recorded with participants’ permission for any subsequent analysis.

The weekly update consultations were planned (date and time) by the parents and principal pediatrician. The consultations between the parents and the pediatrician were conducted in a single-family room. In these private rooms, parents could stay over and sleep next to their infant’s bed. The researcher observed the consultation while simultaneously typing along a basic synopsis of the conversation, noting the questions asked by either the parents or the pediatricians, and the themes that were discussed. A theme checklist was created during the observations: once a theme was brought up, it was written down. If this theme appeared again in another consultation, it was marked in the checklist. The person (mother, father, or pediatrician) who raised a question or theme was also marked for each theme.

After the consultation, parents were asked to complete a questionnaire assessing basic demographic characteristics and their smartphone use. The researcher would leave to give the parents some time to complete the questionnaire. After approximately 15 minutes, the researcher returned to the single-family room to interview the parents. The interviews were audio recorded, and the researcher simultaneously typed the answers. The length of the interviews ranged from 10 to 40 minutes, depending on how elaborate the parents’ answers were. None of the parents declared feeling hindered by the researcher during the consultation. The pediatricians were interviewed either between the consultation and the parents’ interview or after the interview with the parents, depending on whether the pediatrician was busy.

### Analysis

#### Observations

Immediately after the observation, the audio recording and basic synopsis of the consultation were checked alongside the theme checklist for possible missed themes. The questions that were written down in the synopsis were highlighted and classified as asked by the mother, father, or pediatrician. All observational data (demographics, questionnaire information, themes, and questions asked) were interpreted using Microsoft Excel.

#### Interviews

The qualitative interview data were analyzed according to Halcomb and Davidson [24]. First, a basic interview overview was created by writing along a basic synopsis throughout the interview, while the interviews were also being audio recorded. Then, immediately after the interview, the researcher reflected on the data collected in the interview. Third, the interview overviews were evaluated together with the audio recordings to create an accurate interview summary. Relevant information was transcribed precisely into the interview summary accordingly. After completing this process, all the final interview summaries were printed. Using a color-coding system, relevant interview answers of the parents were highlighted and categorized. Conclusions were accordingly drawn from these files.

### Ethical Considerations

On the basis of the nature of the study being an explorative with noninvasive questionnaires the Scientific Board Elisabeth Tweesteden Ziekenhuis declared that the study did not require ethics approval. The study followed all good clinical practice guidelines.

## Results

### Part 1: The Consultation

#### Themes and Questions

In the 19 consultations that were observed, 32 different themes arose (see Table 2 for an overview and explanation of the content). The most frequent topics included feeding and breastfeeding (n=17), introductions of oneself (n=14), parents’ personal life (n=14), criteria for discharge (n=13), and follow-up after discharge (n=13). Except for feeding or breastfeeding and parents’ personal life, most themes were brought up by the pediatrician. The themes most frequently initiated by the parents, included the road to labor (n=7), infant feeding problems (n=7), infant vomiting (n=7), and growth in terms of height (n=7).

With regard to the number of questions asked during the consultations by the parents and pediatricians (RQ2), questions asked by the pediatrician ranged from 0 to 11 care-related questions per consultation, with a mean of 3.7 (SD 2.9) questions per consultation. In sum, the pediatricians had 71 questions for the parents. Mothers (n=19) asked 80 questions (0-15 questions per consultation, with a mean of 4.2, SD 4.3). Fathers (n*=*15) asked 37 questions with a mean of 2.5 (SD 1.9) questions per consultation. Pediatricians explicitly invited parents to ask their questions by asking if they had “any further questions?” in all but 1 (PC03) consultation.

Additional demographic information (Table 1) of the parents was analyzed to determine whether the number of questions from either parents or pediatricians was influenced by aspects such as parents’ age, educational level, how many children they had in total, the health of their infant, the gestational age of their infant, the amount of time spent in the hospital, or the duration of the consultation. However, no noticeable trends were discovered concerning any of these aspects.

The interview data showed that all (PCs; n=19) thought that they had sufficient opportunities to ask questions and were satisfied with the pediatrician’s answers. Six PCs mentioned that they had sometimes forgotten to ask certain questions during the consultation. However, in their explanations, they attributed the reason for this happening to personal reasons (eg, PC 21: “Dad is often not present so he comes up with questions later”). Six PCs stated that if they had forgotten to ask something during the consultation, they would just go and ask the nurses:

Mother: Sometimes we are like ‘we should’ve asked that’ but then we just ask those questions to the nurses, in many cases they know the answers.

PC13

Father: The nursing staff is our go-to resource. The nursing staff often has the answers to the questions we typically ask the pediatrician. Sometimes they have to consult with the pediatrician but we get answers soon after their consultation.

PC13

**Table 2:** Content of the themes and the total number of times a theme was brought up by the pediatrician or the parents for all 19 consultations.

Theme	Content	Values (n=19), n (%)	Introduced by pediatrician^a^, n (%)	Introduced by parents^a^, n (%)
Feeding and breastfeeding	Progression of breastfeeding, milk expression, positive effects	17 (90)	9 (53)	8 (47)
Introduction	Pediatrician introduces oneself; their tasks; the department; daily program	14 (74)	14 (100)	0 (0)
Personal	Personal information about the parents and their lives like work status and psychosocial situation	14 (74)	7 (50)	7 (50)
Criteria	Criteria for discharge	13 (68)	11 (85)	2 (15)
Postdischarge follow-up	Explanation how postnatal follow-up will be scheduled	13 (68)	9 (69)	4 (31)
Respiratory support and caffeine	Supplemental oxygen and pressure, and how these are decreased; medication like caffeine	12 (63)	10 (83)	2 (17)
Not care related	Small talk	11 (59)	4 (36)	7 (64)
Monitor or alarms	Why and for how long the infant will be connected to the monitor; alarms that went because of low oxygen saturation or low heart rate	10 (53)	6 (60)	4 (40)
Department	Opinion and experiences of parents regarding the department; the employees; the nurses; the care; other parents	10 (53)	4 (40)	6 (60)
Talking about the future	The preterm’s future; how will they grow up, which problems may they face, school	8 (42)	6 (75)	2 (25)
Weight	The weight or growth of the preterm infant.	8 (42)	5 (62)	3 (38)
Tube feeding	Whether the preterm is or will be given tube feeding; how much; how long	8 (42)	5 (62)	3 (38)
Temperature of the child	Can the preterm keep themselves warm; external source for heating; incubator; phototherapy for icterus	8 (42)	6 (75)	2 (25)
Ultrasound or MRI^b^ of the brain	Ultrasound or MRI of the brain; and results of them	8 (42)	6 (75)	2 (25)
Vaccinations	Parent’s consent for vaccinations and scheduling	8 (42)	6 (75)	2 (25)
Birth	The process, experience, and possible complications of labor	7 (37)	1 (14)	6 (86)
Vomiting	If and why the preterm is vomiting (explanations)	7 (37)	1 (14)	6 (86)
Height and growth	The length, weight, and growth charts	7 (37)	1 (14)	6 (86)
Laboratory values	blood draw; blood transfusions	7 (37)	7 (100)	0 (0)
How to care at home	After discharge; what should be paid attention to; differences and similarities from raising a term baby and extra concerns	7 (37)	2 (29)	5 (71)
Recap	A recap is given of everything that has happened at the NICU^c^ so far	6 (32)	6 (100)	0 (0)
Emotional	The emotional state of the parents regarding the situation; their preterm; the hospital	6 (32)	5 (83)	1 (17)
Bowel movements	Bowel movements of the preterm; use of enemas	6 (32)	3 (50)	3 (50)
Vitamins	The needed vitamins; iron supplementation	6 (32)	4 (67)	2 (33)
Child specific	Issues specific for the preterm	6 (32)	4 (67)	2 (33)
Data or counting	How the birth date and age of the preterm should be corrected for regarding growth, and scheduling of vaccinations	5 (26)	3 (60)	2 (40)
Infections	Recent infections; chance of infections; antibiotics	5 (26)	5 (100)	0 (0)
Eyes (retinopathy)	Explaining why the eyes are checked; ophthalmologist appointments	4 (21)	1 (25)	3 (75)
NICU level III experience	How parents experienced their stay at the level III NICU; what happened there before transfer	4 (21)	1 (25)	3 (75)
After the NICU	Aftercare by the NICU department; what, when, and how	3 (16)	1 (33)	2 (67)
(Possible) operations	Possible operations of the heart, lungs, brain, body are discussed and explained	2 (11)	1 (50)	1 (50)
Lungs	Explaining pulmonary edema; lung capacity	2 (11)	1 (50)	1 (50)

^a^Total (n)=252. Introduced by pediatrician=155; introduced by parents=97.

^b^MRI: magnetic resonance imaging.

^c^NICU: neonatal intensive care unit.

#### Consultation Evaluation

How parents value the weekly consultation with their principal pediatrician was also studied given that the parents see many different medical professionals daily (RQ3). Overall, the data showed that most PCs needed consultation with their principal pediatricians (n=15). There were 3 overarching reasons parents valued those consultations: finally getting a clear overview of everything (n=6), nice to be kept up to date (n=6), and finally having an interaction with the principal pediatrician (n=3). An example of the latter category is presented in the following quote:

Yes, we had a great need actually. We actually had some of our questions answered during rounds, but that moment is fairly short. That was also explained to us. For certain questions, they make sure we get moments like this [the weekly meeting with the main pediatrician]. This was a great moment. We had many questions about what will happen when we leave. These came forth from changes that have been happening. Those things all come together now and we just want to know what will happen next week.

PC14

Four PCs had a lesser need for weekly consultations. These parents stated that the consultation did not provide extra information because they had already received information from nurses or other professionals during their daily rounds. However, 1 PC (as noted in the aforementioned quote) did have a need for the weekly meeting with the pediatrician despite having received information during daily rounds. Thus, valuing the consultation seems to depend on the parents’ perspectives and experiences.

Almost all parents were pleased with how the different professionals of the neonatal unit communicated. Repeatedly, parents gave short answers if they were satisfied with the level of communication (eg, PC06: “Yes, very satisfied with the communication”). The parents unanimously declared to have felt included and stated that no decisions were made without consulting the parents first. PCs that were slightly less positive (n=4) shared the same overarching reason for their opinion: the medical staff members’ team is large, and there are many different professionals who have their own way of communicating and caring. This opinion is best summarized in the following statements:

In general, the communication is very good. Yes, I know that for sure. However, you do notice that everyone has his own method of caring for our baby. And ehm...that is a disadvantage because it is a large team. You get acquainted with someone who is taking care of us, you just get to know someone and their ways and then the next person arrives. You keep switching between how people do everything.

PC10

I do however notice that when another nurse takes over something, that I can just pick up where I left off with everything that I was saying. I don’t have the idea that I have to fill in certain blanks.

PC20

### Part 2: Parents’ and Pediatricians’ Interest in an mHealth App

#### Parents’ Need for an mHealth App

Next, we examined whether the parents would appreciate a communication and question-asking mHealth app that could be used during the hospitalization period (RQ 4, 5, and 6). This hypothetical app could give parents the opportunity to disclose questions, themes, and concerns to the pediatrician days before the consultation.

In total, 17 of the 19 PCs’ valued the idea of this type of app, 10 of whom indicated that they would definitely use it; 8 of these couples mentioned that they believe it is practical to be able to write down their questions as soon as they think of one:

Sometimes you think of questions when you are not in the room [and do not have anything with you to take notes] and you of course, always have your phone with you [...]. Just easier when you have an app, especially now that we live in a mobile era.

PC19

Yes, I think it has value [the app]. You can, the moment you think of a question, write it down. That might bring you peace.

PC05

Seven couples liked the idea of the app but did not believe they would use it themselves. In explaining their point of view, 2 PCs (PC09 and PC14) mentioned that they could, however, understand the benefits of the app for medical professionals:

Well, not that much [need for such an app for us] actually. I can imagine there are people who would have a need for such an app but I don’t think we would use it that often [...] I do think it is a good initiative. There is a low threshold to present certain problems to the doctor and the doctor can then focus on the needs of the parents and prepare themselves better [...]. And, as a doctor you get to say “I have to come back to that later” less often, in terms of efficiency it is very useful. Yes, I am positive about an app, not necessarily for us, but I do believe that parents can benefit from this. Especially new parents.

C14

Overall, 14 of the 19 PCs stated that using such an app could probably change the way they ask questions. Parents provided various reasons for this. For example, most (n=10) PCs mentioned that they would probably ask more questions because they were less likely to forget them. Four parents stated that given the potential features of the mHealth app, they would probably have more questions because they had access to more information:

If there would be, for instance, a questions suggestions list, then I would probably ask more questions. Or certain information about preterm infants in general, which I haven’t personally thought about yet, then I would think like “hey I would want to know something about that.”

PC11

Four PCs stated that they would probably ask fewer questions if the app contained basic medical information about preterm infants. One PC (C10) mentioned that they would probably ask more in-depth questions during the consultation because their questions have already been made clear to the caregiver beforehand. This could make consultations elapse more efficiently and satisfactorily.

The motivations of the 2 PCs (PC06 and PC12) who did not need the app were similar. They preferred real-life contact over all else and were not, in their words, the “app kind of people.”

An overview of parents’ ideas for the mHealth app is presented in Table 3 (RQ4). The most mentioned feature by the parents was a graph feature, which included their infants’ growth. Most parents valued this function because of entertaining grounds (eg, C14: “something like a weight tracker is fun”), whereas others valued this feature because of their need to keep track of their infant’s progress (eg, PC05: “keeping track of their progress would be nice [by using trackers], I keep track of everything myself now but in an app would be nice, clear”).

Parents who valued a diary or achievements feature had similar reasons for why they would like such a feature: either because of entertaining properties or because they wanted to keep track of their infants’ progress. One of the PCs proposed the option of having a list to mark off specific milestones (eg, weight >2000 g) in a playful way as part of the diary function. They believed that this would help keep the parents motivated in working toward discharge from the hospital.

**Table 3:** Features, the content of features, and frequency of interviews the feature was mentioned in.

Features	Content	Frequency of interviews in which the feature was mentioned (n=19), n (%)
Graphs	Graphs including their infants’ growth or weight and what is normal for infants to grow or weigh (comparisons)	8 (42)
Diary or achievements	Diary in which parents could write their own experiences and condition of their infant	6 (32)
Information	Practical basic information, glossary of terms, what to expect with a preterm infant	6 (32)
Medical rounds reports	Report of what is discussed during medical rounds provided by all medical caregivers, including the current values	5 (26)
Baby cam	Camera feature that shows their infant in their bed at the hospital	5 (26)
Agenda	Schedule including planning or appointments	4 (21)
Frequently asked questions	Frequently asked questions with answers	3 (16)
Question suggestions	Suggestions on what the parents could ask their caregivers	2 (11)
Pictures	Saving and sending pictures	2 (11)
Information medical exams	Explanation of different medical exams	1 (5)
Results medical exams	Results of the exams (eg, blood testing, MRI^a^)	1 (5)
Parental presence	Giving parents the option to state when they are at the hospital and when they are gone	1 (5)

^a^MRI: magnetic resonance imaging.

The medical rounds report feature is the final feature worth mentioning. During medical rounds in the morning, general care-related information about the infant is discussed, as well as information such as what happened during the night [9]. Several parents indicated that they saw medical caregivers typing along the discussed information during medical rounds and would also like to have that information:

During rounds you see the doctor typing little reports, you remember it [the information discussed] as a parent but it is still nice to check it later on. Sometimes I forget the easy things like their weight. Just some broad outlines I would definitely like to have. Also, maybe if later on something happens that you are able to see that back.

PC20

The main reason some PCs wanted these reports was that they simply wanted to be able to reread the information.

#### Pediatricians’ Need for an mHealth App

The pediatricians (n=7) were also asked how they would feel about using the hypothetical mHealth app (RQ6). All pediatricians were positive about the idea of a communication app. Four of 7 pediatricians believed the app could personally help them in being better prepared for the consultation because of the potential app’s function to read parents’ concerns and questions beforehand. Three of the 7 pediatricians thought that the app may be particularly helpful for the more anxious parents by having them store questions and concerns at any moment at any place, thereby relieving some stress:

Yes [I would value such an app] because you can see what the parents are occupied with beforehand so you can already prepare yourself for the questions they are having instead of having to get back to it during medical rounds.

D06

It would be nice if I was able to check the old-fashioned lists parents normally bring to the consult. Imagine that they would ask something I wouldn’t know the answer to, for instance “when is the appointment with the eye doctor?”, then I could look this up beforehand and would be able to have a more efficient interaction. For that matter, there are also questions that you of course expect [...] however, unexpected questions do happen and having that [such an app] would be nice to have.

D03

## Discussion

### Principal Findings

The interaction between the pediatrician and parents, as well as their digital communication technology needs, were evaluated in a 2-part exploratory needs assessment, with the goal of improving interpersonal communication and information exchange. The results from the first part of the study showed that pediatricians introduced most themes, of which feeding and breastfeeding, personal introductions, the consultation office, medication (caffeine), and parents’ personal lives were the most frequent ones.

Themes initiated by the medical caregivers such as introducing themselves or asking the parents about their personal lives are not medical themes but topics that reflect a caring environment in the neonatal department [23]. The pediatricians seem to be engaged in ensuring that parents understand the hospital situation and all staff members. This is also demonstrated by the pediatricians’ concern for the parents’ questions and satisfaction with the communication. However, there was 1 aspect that received some criticism from multiple parents, namely, the constantly shifting nursing staff. This is not a novel result and is difficult to change [5]. However, most of the parents stated that although the nursing staff is always changing, all the nurses are up to date on how the parents and the infant are doing, and transitioning between nurses is relatively easy.

Regarding question asking, observational results showed that mothers asked more questions than fathers, and the majority of the PCs stated that they did not forget to ask certain questions during the consultation. This answer could potentially be explained by having the interview conducted shortly after the consultation. Given that the parents were still processing the information from their appointment with the pediatrician [10], it could be that parents did not realize that they had forgotten to ask a question. Fortunately, the interview results show that parents experience a hospital environment in which they are able to ask questions to various medical caregivers 24/7. Several parents explained that if they forgot to ask something or came up with a question randomly, they just asked the nurses. Finally, although parents are going through difficult and stressful times while they are in the hospital [6], they were all quite optimistic during the interview and talked highly of the neonatal unit and its staff.

Then, the second part of our study used the mHealth app. Nearly all parents seemed to value the idea of the mHealth app and recognize the possible benefits it would have for them. Providing information has been the main purpose of most existing neonatal mHealth apps, and this study has shown that the same remains an important feature of an mHealth intervention [12]. In essence, almost all features frequently mentioned by the parents are linked to retrieving information. Some parents expressed this by simply desiring a standard information feature, some by wanting the reports from the medical rounds, whereas others valued the most frequently asked questions, and so on. This resembles what is depicted in the literature regarding the needs of parents of preterm infants: parents have extensive information needs and crave to satisfy these needs by seeking information from various sources [11,12,15].

Previous studies have repeatedly shown that patients and physicians value different aspects of supporting interventions, and this study supports that notion [20]. For example, during the observations, the parents often initiated the theme “increase in length,” whereas the pediatricians were not concerned about the length because, from a medical standpoint, weight gain is much more important than increase in length. The length was also the most frequently mentioned app feature that parents would find valuable. This illustrates that there seems to be a discrepancy between what parents want to know and what is important for professionals [20]. Furthermore, it supports the idea that it is important to include all the different stakeholders in developing an mHealth app [19].

According to parents, the actual use of the hypothetical app will depend on a variety of personal factors, such as their general opinion of electronic interventions or how difficult or easy the hospital stay is. These are personal experiences and points of view that are likely to be expressed in connection with other possible electronic interventions and are not necessarily related to a particular mHealth app’s content.

In summary, even though parents are positive about the consultations, most parents still value using an mHealth app because they have a strong need for information and a desire to feel deeply involved in the entire process [6]. The proposed mHealth app mostly received good feedback from both parents and pediatricians.

### Theoretical and Societal Implications

This study has several theoretical and practical implications. First, to our knowledge, this study is one of the few studies to observe how consultations organically unfold at a level II neonatal unit [18]. The findings from the observations have provided a comprehensive list of themes that are discussed during consultations in addition to who initiates these themes, allowing for insights into the topics that both pediatricians and parents deem crucial to discuss. This could help medical caregivers guide their consultations. In addition, by supplementing observational data with interview data, a comprehensive understanding of the current interactions and how parents felt about the consultations was obtained.

Furthermore, this study is one of the few to our knowledge to use scientific research to gather insights for the creation of a neonatal mHealth app and to examine parental perceptions before app development, which will hopefully boost the possibility of creating an appropriate intervention [22]. Although the major purpose of the mHealth app was to give parents the option of asking their caregivers questions ahead of time, our interview data revealed that parents wanted several other functionalities. In addition, for other neonatal mHealth developers, we recommend that at least the following features are present in such an app: (1) graphs about the child’s growth with the option of comparing and checking milestones, (2) information including frequently asked questions that resemble the information a hospital provides because parents value hospital-specific information instead of general preterm information, and (3) a summary of the daily medical rounds.

Furthermore, based on our findings, we were able to make some concrete recommendations that mHealth app developers, in general, may consider when designing an mHealth app. Of course, different mHealth apps require various recommendations. However, the recommendations in Textbox 1 are what we advise for comparable apps.

**Recommendations for mHealth developers.**

- Future users should be involved early in the app’s development process.
- The app should have an attractive nonmedical look and feel.
- The app should be a useful tool, it should not replace aspects such as general information provision, sharing specific medical data, or planning.
- The app should not be intrusive for medical caregivers. It should not disturb daily work nor add to the workload of the caregivers.

### Limitations and Suggestions for Future Research

Our findings must also be viewed in light of these limitations. First, these premature infants were not critically ill, which rendered positive and hopeful consultations that could have positively influenced their parents. Nevertheless, there is not necessarily a reason to believe that pediatricians will behave differently in consultations when the infant is in a more critical health condition. To be able to evaluate the impact of the preterm infant’s health on the caregiver-parent dialogue, future research on this topic should therefore include a more diverse patient population.

The next limitation is that the sample was taken from 2 Dutch hospitals. It could therefore be argued that our results lack generalization because every country has its own culture and also its way of communicating. Our data reflect Dutch neonatal structures and ways of communication. Future research should examine and compare the communication at more neonatal units internationally to obtain a clearer picture of the current communication structures.

Finally, although parents were satisfied with the consultation, we did not examine whether the physicians’ information was fully understood by the parents. Research has shown that parents tend to have difficulty understanding what is being said [4,25]. As correct parental understanding is also highly important for navigating the emotional hospital period and time thereafter [4], we propose future research on this topic.

### Conclusions

On the basis of the results from both the observations and interviews, it is possible to conclude that, according to parents, communication at the neonatal unit is effective, informative, and satisfying. The second part of the study showed great interest in the potential mHealth app by both the parents and the pediatricians and yielded interesting insights for the development of mHealth apps.

This explorative study is a foundation for things to come. This study has paved the way for the next step in creating the communicative mHealth intervention in which there will still be plenty to be discovered regarding the app and its potential. We believe that an mHealth intervention has potential benefits for both parents and medical professionals. Hence, continuing research and development of mHealth apps is encouraged.

## Abbreviations

mHealth: mobile health
NICU: neonatal intensive care unit
PC: parent-couple
RQ: research question

## References

[1] Bakewell-Sachs S, Gennaro S (2004). Parenting the post-NICU premature infant. MCN Am J Matern Child Nurs.

[2] Jackson K, Ternestedt BM, Schollin J (2003). From alienation to familiarity: experiences of mothers and fathers of preterm infants. J Adv Nurs.

[3] Mäkelä H, Axelin A, Feeley N, Niela-Vilén H (2018). Clinging to closeness: the parental view on developing a close bond with their infants in a NICU. Midwifery.

[4] Wigert H, Dellenmark Blom M, Bry K (2014). Parents' experiences of communication with neonatal intensive-care unit staff: an interview study. BMC Pediatr.

[5] Lantz B (2017). Information to parents in the neonatal unit. J Neonatal Nurs.

[6] Kowalski WJ, Leef KH, Mackley A, Spear ML, Paul DA (2006). Communicating with parents of premature infants: who is the informant?. J Perinatol.

[7] Griffin T (2006). Family-centered care in the NICU. J Perinat Neonatal Nurs.

[8] Hagen IH, Iversen VC, Nesset E, Orner R, Svindseth MF (2019). Parental satisfaction with neonatal intensive care units: a quantitative cross-sectional study. BMC Health Serv Res.

[9] Hill C, Knafl KA, Santacroce SJ (2018). Family-centered care from the perspective of parents of children cared for in a pediatric intensive care unit: an integrative review. J Pediatr Nurs.

[10] Aronson PL, Yau J, Helfaer MA, Morrison W (2009). Impact of family presence during pediatric intensive care unit rounds on the family and medical team. Pediatrics.

[11] Spargo P, de Vries NK (2018). 'Babble': a smartphone app for parents who have a baby in the neonatal unit. J Paediatr Child Health.

[12] Richardson B, Dol J, Rutledge K, Monaghan J, Orovec A, Howie K, Boates T, Smit M, Campbell-Yeo M (2019). Evaluation of mobile apps targeted to parents of infants in the neonatal intensive care unit: systematic app review. JMIR Mhealth Uhealth.

[13] Van Dijk MR, Koster MP, Rosman AN, Steegers-Theunissen RP (2017). Opportunities of mHealth in preconception care: preferences and experiences of patients and health care providers and other involved professionals. JMIR Mhealth Uhealth.

[14] Garfield CF, Lee YS, Kim HN, Rutsohn J, Kahn JY, Mustanski B, Mohr DC (2016). Supporting parents of premature infants transitioning from the NICU to home: a pilot randomized control trial of a smartphone application. Internet Interv.

[15] Shorey S, Ng YP, Danbjørg DB, Dennis CL, Morelius E (2017). Effectiveness of the 'Home-but not Alone' mobile health application educational programme on parental outcomes: a randomized controlled trial, study protocol. J Adv Nurs.

[16] Dol J, Delahunty-Pike A, Anwar Siani S, Campbell-Yeo M (2017). eHealth interventions for parents in neonatal intensive care units: a systematic review. JBI Database System Rev Implement Rep.

[17] Doron MW, Trenti-Paroli E, Linden DW (2013). Supporting parents in the NICU: a new app from the US, ‘MyPreemie’: a tool to provide parents of premature babies with support, empowerment, education and participation in their infant's care. J Neonatal Nurs.

[18] Boss RD, Donohue PK, Larson SM, Arnold RM, Roter DL (2016). Family conferences in the neonatal ICU: observation of communication dynamics and contributions. Pediatr Crit Care Med.

[19] Nievas-Soriano BJ, García-Duarte S, Fernández-Alonso AM, Bonillo-Perales A, Parrón-Carreño T (2021). Users evaluation of a Spanish eHealth pediatric website. Comput Methods Programs Biomed.

[20] Garne K, Brødsgaard A, Zachariassen G, Clemensen J (2016). Telemedicine in neonatal home care: identifying parental needs through participatory design. JMIR Res Protoc.

[21] Miller N, Rogers SN (2018). A review of question prompt lists used in the oncology setting with comparison to the Patient Concerns Inventory. Eur J Cancer Care (Engl).

[22] Francis JJ, Johnston M, Robertson C, Glidewell L, Entwistle V, Eccles MP, Grimshaw JM (2010). What is an adequate sample size? Operationalising data saturation for theory-based interview studies. Psychol Health.

[23] American Academy of Pediatrics Committee on Fetus And Newborn (2012). Levels of neonatal care. Pediatrics.

[24] Halcomb EJ, Davidson PM (2006). Is verbatim transcription of interview data always necessary?. Appl Nurs Res.

[25] Payot A, Gendron S, Lefebvre F, Doucet H (2007). Deciding to resuscitate extremely premature babies: how do parents and neonatologists engage in the decision?. Soc Sci Med.

